# Redox regulation of LSD1/CATALASE 2 phase separation condensates controls location and functions

**DOI:** 10.1111/nph.70374

**Published:** 2025-07-21

**Authors:** Chi‐Chuan Lin, Christine H. Foyer, Megan Wright, Alison Baker

**Affiliations:** ^1^ School of Molecular and Cellular Biology, Centre for Plant Sciences and Astbury Centre for Structural Molecular Biology University of Leeds Leeds LS2 9JT UK; ^2^ School of Biosciences, College of Life and Environment Sciences University of Birmingham Edgbaston B15 2TT UK; ^3^ School of Chemistry, Faculty of Engineering and Physical Sciences, and Astbury Centre for Structural Molecular Biology University of Leeds Leeds LS2 9JT UK

**Keywords:** *Arabidopsis thaliana* catalase, biomolecular condensates, LSD1, nucleus, peroxisome, protein trafficking, redox

## Abstract

- Phase separation of proteins into membraneless compartments is emerging as an important mechanism of plant developmental and stress responses. We show Arabidopsis catalase 2 (CAT2) is recruited to phase‐separated condensates with LESION SIMULATING DISEASE1 (LSD1), a plant‐specific regulator of programmed cell death, in a redox‐dependent manner that regulates its intracellular localisation and activity.
- Using recombinant proteins, we showed that CAT2 and LSD1 form ternary complexes with the peroxisome import receptor PEX5. The ability of LSD1 to form phase‐separated condensates is a property of zinc fingers 1 and 2. The interactions between all three proteins and the fluidity of the LSD1 condensates are redox‐regulated.
- Using confocal microscopy, the *in vivo* trafficking of CAT2 to peroxisomes and the nuclei was shown to be redox‐regulated, and LSD1 was shown to control CAT2 localisation *in vivo*.
- We propose a model whereby the redox‐dependent differential accessibility of CAT2, PEX5 and LSD1 within condensates not only regulates CAT2 activity but also compartmentalisation between peroxisome, cytosol and nucleus. Relocation of catalase to the nucleus may provide protection to nuclear processes under conditions of biotic stress.

## Introduction

The redox environment of plant cells regulates every aspect of growth, development and defence, including the transcription factors and enzymes involved in genetic and epigenetic changes (Auverlot *et al*., 2024). Within this context, the ability of cells to enhance antioxidant enzyme capacity is an essential facet of adaptive oxidative responses to environmental challenges. Many proteins have been shown to separate from the dilute, soluble phase through phase separation, forming membraneless organelles or condensates through protein–protein interactions (Banani *et al*., 2017). Such condensates play versatile roles in the sensing and scavenging of reactive oxygen species to maintain cellular redox homeostasis in animals (Saito & Kimura, 2021). In recent years, it has emerged that the formation of biological condensates also regulates key processes in plants (Emenecker *et al*., 2020, 2021; Wang & Gu, 2022). However, biological condensate research in plants is still in its infancy (Q. Liu *et al*., 2024). The redox‐mediated regulation of development is highlighted by the cysteine oxidation‐dependent control of the formation of condensates of the TERMINATING FLOWER (TMF) transcription factor that determines the flowering transition in the tomato shoot apical meristem (X. Huang *et al*., 2021). Guanylate binding protein‐like GTPases undergo phase transition to regulate transcriptional responses involved in plant immunity (S. Huang *et al*., 2021). The NONEXPRESSOR OF PATHOGENESIS‐RELATED GENES 1 (NPR1) protein, which is an important transcription factor regulating systemic acquired immunity, also forms condensates to regulate plant cell death responses (Zavaliev *et al*., 2020). Phase separation of the central barrier of the nuclear pore complex regulates nucleocytoplasmic transport of immune regulators (Wang *et al*., 2023). Reactive oxygen species production and redox processing are central mechanisms in the regulation of plant growth and responses to environmental stresses (C. Liu *et al*., 2024). While phase separation is established as a mechanism that regulates plant development and immunity, its role in controlling the redox state of plant cells remains to be established.

Catalases are important antioxidant enzymes that regulate levels of H_2_O_2_ in cells (Baker *et al*., 2023). Catalase is predominantly localised to the peroxisomes in eukaryotes, although cytosolic pools have been reported (Mhamdi *et al*., 2012). Catalase is imported by the major peroxisomal PTS1 import receptor PEX5 but via a so called ‘noncanonical’ pathway (Rymer *et al*., 2018) and the cycling of the PEX5 receptor between cytosol and peroxisome is also sensitive to cellular redox state via the oxidation of a conserved cysteine in the N terminus of PEX5 (Apanasets *et al*., 2014). In *Arabidopsis thaliana*, there are three isoforms of catalase with different expression patterns (Frugoli *et al*., 1996). CAT2 accounts for *c*. 80% of total activity in leaf (Queval *et al*., 2010). Despite being predominantly localised in peroxisomes, plant catalases have an ever‐expanding list of protein interactors, most of which are cytosolic proteins (reviewed in Foyer *et al*., 2020). These include endogenous plant proteins (Zhou *et al*., 2018) and pathogen effectors (Zhang *et al*., 2015), the latter study showing that these effectors could traffic catalase to the nucleus to manipulate cell survival. A re‐examination of catalase targeting *in planta* using a split Green Fluorecent Protein (GFP) system provided evidence for nuclear‐localised catalase in uninfected cells (Al‐Hajaya *et al*., 2022). Collectively, these observations point to the redirection of catalase activity and location as a potential mechanism for controlling redox processing and signalling (Baker *et al*., 2023).

One of the endogenous interacting proteins of catalase is LESION SIMULATING DISEASE1 (LSD1) (Li *et al*., 2013), which itself can interact with a wide range of binding partners in a redox‐dependent manner (Czarnocka *et al*., 2017). LSD1 is a plant‐specific protein that participates in the control of redox signals to suppress programmed cell death (PCD). The *lsd1* mutants show a runaway cell death phenotype (Jabs *et al*., 1996). LSD1 is localised in the nucleus and cytosol, where it acts as a scaffold protein and a transcriptional regulator/coregulator (Czarnocka *et al*., 2017; Li *et al*., 2022). LSD1 can modulate the activity of the bZIP10 transcription factor, a positive regulator of the hypersensitive response and PCD by preventing the movement of this protein into the nucleus (Kaminaka *et al*., 2006).

In this study, we show that LSD1 forms biomolecular condensates to recruit CAT2 and upregulate its activity *in vitro*, and that the interactions between CAT2, LSD1 and PEX5 are redox‐sensitive. *In vivo*, LSD1 colocalises with CAT2; moreover, CAT2 is mislocalised in the *lsd1* mutant. The redox‐dependent control of CAT2 and LSD1 localisation and function has important implications for redox signalling under stress conditions.

## Materials and Methods

### Molecular cloning

For recombinant protein production, the CAT2 gene fragment was amplified and cloned into a pET28b vector using NdeI and XhoI. LSD1 (full‐length and individual domains) fragments were amplified and cloned into a pET28b vector using NdeI and NotI.

### Protein expression and purification

6×Histidine‐tagged LSD1, PEX5 and CAT2 proteins were expressed and purified from BL21(DE3) cells. Ten millilitres of cells grown overnight were used to inoculate 1 l of LB media with 50 μg ml^−1^ kanamycin and grown with constant shaking (200 rpm) at 37°C until the OD_600_ = 0.8. At this point, the culture was cooled to 20°C before being induced by the addition of 0.1 mM IPTG (BP1755100; Fisher Scientific UK Ltd, Loughborough, UK). The culture was allowed to grow for a further 12 h at 20°C before harvesting by centrifugation. Cells were resuspended in 50 mM Tris buffer at pH 8.0, containing 300 mM NaCl, in the presence of protease inhibitors (1 tablet for a 10 ml cell suspension, 11 836 170 001; Sigma) and lysed by sonication. Cell debris was removed by centrifugation (20 000 ***g*** at 4°C for 60 min). The soluble fraction was applied to an affinity column previously loaded with the above buffer followed by washing in 20 mM imidazole. Protein was eluted from the column with 200 mM imidazole.

LSD1 protein was dialysed into a 20 mM HEPES buffer at pH 7.2 or 8.3, containing 20 mM NaCl. PEX5 and CAT2 proteins were further concentrated to 5 ml and applied to a Superdex SD200 or a Sephacryl S‐100 gel filtration column equilibrated with 20 mM HEPES buffer at pH 7.2 or 8.3, containing 20 mM NaCl. Analysis of pure proteins on SDS‐PAGE (Biorad Mini‐PROTEAN TGX Precast Protein Gels, 4561 093, 4561 096, 4561 034 and 4561 036) showed > 95% purity using Coomassie staining.

### Microscale thermophoresis

Microscale thermophoresis (MST) was performed as described (Jerabek‐Willemsen *et al*., 2011). The binding affinities were measured using the Monolith NT.115 from Nanotemper Technologies GmbH, Munich, Germany. Proteins were fluorescently labelled with Atto‐488 dye according to the manufacturer's protocol. Labelling efficiency was determined to be 1 : 1 (protein to dye). A solution of unlabelled binding partner was serially diluted and added to 100 nM of labelled protein. The samples were loaded into premium capillaries (Nanotemper Technologies Gmbh) after incubation at room temperature for 10 min. Measurements were performed at 25°C in 20 mM HEPES buffer, pH 7.5, with 20 mM NaCl and 0.05% (v/v) Tween 20. Data analyses were performed using nanotemper analysis software, v.1.2.101.

### Protein fluorescent labelling

Highly purified LSD1, PEX5 and CAT2 proteins (in 20 mM HEPES, pH 7.5 and 20 mM NaCl) were prepared in 100 mM NaHCO_3_ buffer (pH 8.3) at 2 mg ml^−1^ and labelled with Atto‐488 NHS ester (41698; Sigma), Atto‐550 (92835; Sigma) or Atto‐647 NHS ester (07376; Sigma) respectively and incubated at room temperature for 1 h (fluorophore to protein molar ratio was 1 : 1). Excess dye was removed, and buffer was exchanged using a G‐15 desalting column (G15120; Sigma). Proteins were concentrated, and labelling efficiency was measured by Nanodrop 2000 (ThermoFisher Scientific (Life Technologies), Paisley, UK).

### Plant materials and growth conditions

*Arabidopsis thaliana* L. Heynh wild‐type (Col‐0) was from laboratory stocks. The LSD1 knockout line (*lsd1‐2*, CS68738 Salk_042687) (Kaminaka *et al*., 2006) was obtained from the Arabidopsis Biological Resource Center.

Seeds were sterilised in 70% (v/v) ethanol for 5 min, followed by 7% (v/v) bleach solution for 15 min. After washing five times with water, seeds were resuspended in 0.1% (w/v) sterile agarose solution and stratified at 4°C for 48 h. Seeds were sown on ½ Murashige & Skoog media plates and placed for 5 d in a 16 h : 8 h, light : dark photoperiod in a growth chamber. Then, seedlings were transferred into soil and grown for 4 wk in a controlled environment growth chamber (16 h light, 20°C, 60% humidity).

### Preparation of protoplasts

Arabidopsis protoplasts were prepared from leaves of 5‐ to 6‐wk‐old plants grown under controlled conditions and transfected via Polyethylene Glycol (PEG)‐mediated transient transformation according to Wu *et al*. (2009). Plasmid CFP‐SKL is described in the publication (Sparkes *et al*., 2005).

### Immunofluorescence

Protoplasts were seeded on coverslips in a 24‐well plate. Cells were fixed for 15 min with 4% (v/v) paraformaldehyde at 37°C, then washed three times at room temperature with washing buffer (1× TBS and 1% (v/v) Tween‐20) for 10 min each with gentle rocking and incubated in blocking buffer (1× TBS with 3% (w/v) BSA and 1% (v/v) Tween‐20) for 1 h. To investigate the effect of redox conditions on the changes of CAT2 cellular localisation, H_2_O_2_ or β‐ME was added to reach a final concentration of 10 mM and incubated at room temperature for a further 4 h before PFA fixing. Blocking buffer was aspirated and cells were incubated with primary antibody (1 : 200 dilution; LSD1: Agrisera, AS122104; CAT2: PHYTOAB, PHY3044A; monoclonal anti‐CAT2 specific (gift from Dr Changle Ma (Su *et al*., 2018)); PEX14: Rabbit, AS08372, Agrisera; PEX5: Genosphere, Customised, antigen: recombinant Arabidopsis PEX5 amino acids 340–728) as indicated overnight in the dark at 4°C. The following day, cells were washed three times with washing buffer for 10 min each with gentle rocking, incubated with fluorophore‐conjugated secondary antibodies (1 : 1000 dilution; Anti‐chicken secondary‐Alexa 405, ThermoFisher, A‐31556; Anti‐rabbit secondary‐Alexa 488, ThermoFisher, A‐11008; Anti‐rabbit secondary‐Alexa 546, ThermoFisher, A‐11035; Anti‐mouse secondary‐Alexa 488, ThermoFisher, A‐11001) and nuclear stain for 1 h at room temperature in the dark, washed and then mounted. Slides were analysed using a Zeiss LSM 880 inverted or upright confocal microscope, and images were analysed using imagej software.

### Confocal microscopy of purified protein condensates

Phase‐separated condensates of purified LSD1 were formed by dialysis of the purified protein into a buffer containing 20 mM HEPES (pH 7.2 or 8.3) and 20 mM NaCl to reach the final protein concentration of 1–2 mg ml^−1^ at room temperature for 3 h. For the complexes, purified CAT2 and/or PEX5 were added to the LSD1 condensates and incubated for 1 min before imaging. To investigate the effect of redox conditions on condensate, H_2_O_2_ or β‐ME was added to reach a final concentration of 10 mM and incubated at temperature for a further 1 min before imaging. To investigate how 1,6‐hexanediol regulates LSD1 condensate formation, 50% (v/v) of 1,6‐hexanediol stock was added to the sample solution to reach the indicated concentrations. 1,6‐Hexanediol was purchased from Sigma (240117), warmed up to 45°C, and dissolved to a 50%(v/v) stock solution in water. Samples imaged with confocal microscopy were added to glass‐bottom 3‐well chamber slides to image at 40× magnification (oil lens, 1.4 numerical aperture) on a Zeiss LSM880 upright or inverted laser scanning confocal microscope. Proteins were labelled with Atto 488, 550, or 647 dyes as indicated. Images were taken at the indicated times, and proteins were at the indicated concentrations. Image analysis was used to measure the extent of condensate formation, number, size and fluorescence intensity using fiji imagej software.

### Fluorescence recovery after photobleaching (FRAP) assay

The FRAP assay of LSD1 was performed on a Zeiss LSM880 inverted confocal microscope at room temperature. Fluorescent signals were bleached using the appropriate corresponding laser beam. Fluorescence intensity was recorded in two regions: a region that was bleached and a region of equal size that was not bleached. The unbleached region was used as a control for the stability of the fluorescence signal throughout the FRAP experiment. Fluorescence signal from the bleached region was normalised to the unbleached region, which is then expressed as a fraction of the normalised signal before bleaching. The fluorescence intensity at time 0 was normalised to 1.

### Catalase activity assay

Catalase activity is measured by the UV spectrophotometric method, which depends on monitoring the change at 240 nm absorbance when catalase is added to a 30 mM H_2_O_2_ solution. Briefly, samples are added to the H_2_O_2_ solution and incubated at room temperature for 1 min, and the changes in absorbance are measured (Beers Jr. & Sizer, 1952).

## Results

### LSD1 interacts with CAT2 in the presence of PEX5 in solution

**Fig. 1:**
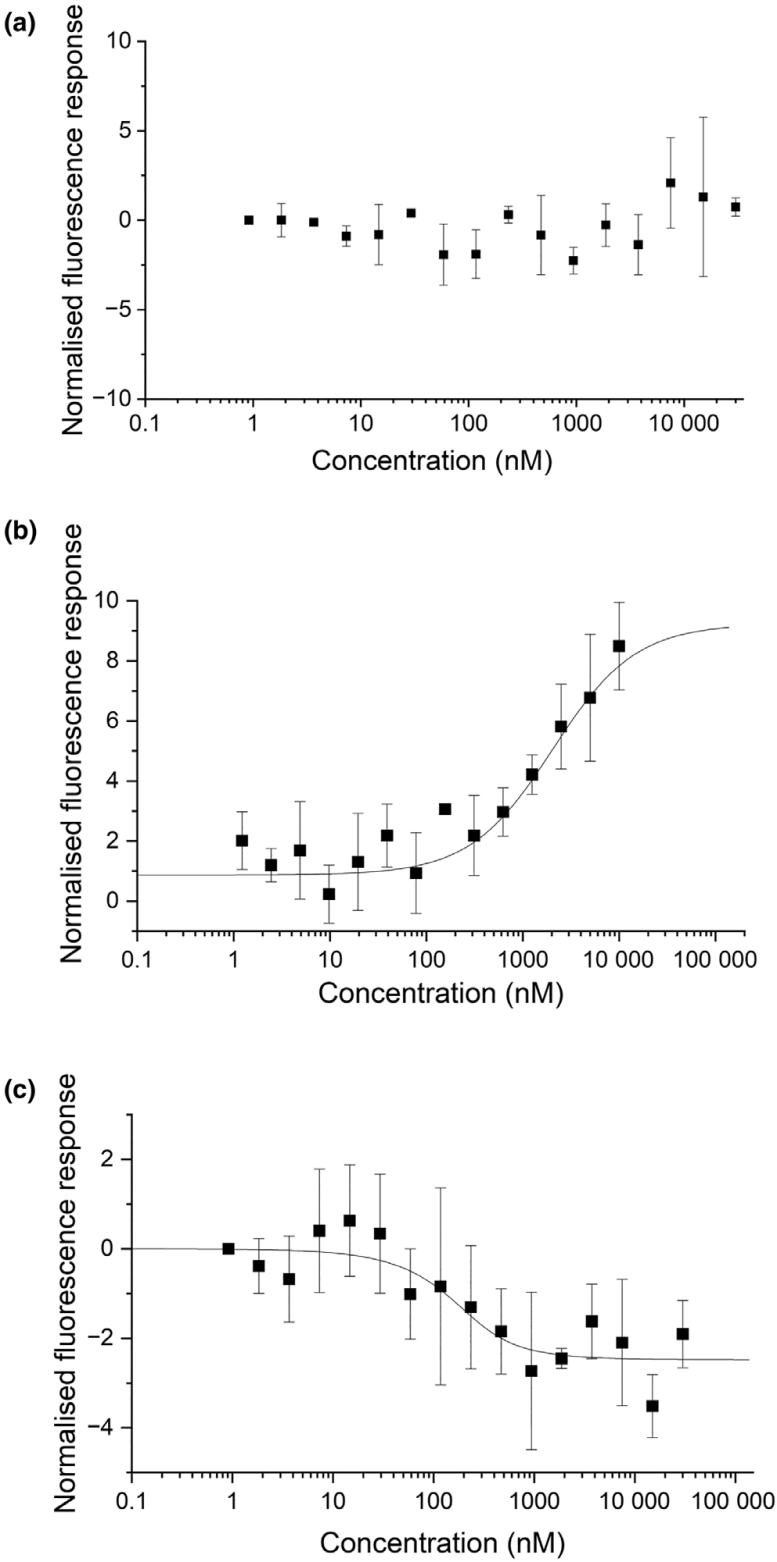
Biophysical analysis of *Arabidopsis thaliana* LSD1 interaction with CAT2 and PEX5. (a) MST characterisation of LSD1 binding to fluorescent labelled CAT2. Data are presented as mean ± SD of technical duplicates. (b) MST characterisation of PEX5 binding to fluorescent labelled CAT2. Data are presented as mean ± SD of technical triplicates. (c) MST characterisation of LSD1 binding to fluorescent‐labelled CAT2 saturated with PEX5. Data are presented as mean ± SD of technical triplicates. MST monitors the movement of molecules in a directed manner within a temperature gradient. The direction of the curves is influenced by the size, charge and solvation shell of the molecules. Therefore, the PEX5–CAT2 complex and the LSD1–CAT2–PEX5 complex show different movement properties.

Catalase was previously reported to bind to LSD1, but to establish whether this binding is direct, MST with recombinant Arabidopsis LSD1 and CAT2 proteins was used, but no significant interaction was detected (Fig. 1a, Supporting Information Fig. S1). By contrast, CAT2 bound to the recombinant Arabidopsis PEX5 protein in the MST assays with an affinity of 2.35 ± 0.279 μM (Fig. 1b). To determine whether LSD1 binds to the PEX5–CAT2 complex, Atto‐488‐labelled CAT2 was saturated with PEX5 (at a concentration 10‐fold above the affinity values) before MST measurements. A clear interaction event was observed upon titrating LSD1, and a sigmoidal curve with an affinity of 0.136 ± 0.0398 μM could be fitted (Fig. 1c), indicating the formation of a ternary complex where PEX5 brings CAT2 and LSD1 together.

### LSD1 forms granule‐like structures in cells and co‐localises with PEX5 and CAT2 and peroxisome markers

Endogenous LSD1 was detected in the nucleus and the cytosol of Arabidopsis protoplasts by immunofluorescence, where it forms granule‐like speckles (Fig. S2ai,ii). The specificity of the anti‐LSD1 antibody is demonstrated by the lack of staining in *lsd1* mutant protoplasts (Fig. S2aiii,iv). CAT2 is one of three very similar isoforms of catalase that are not distinguished by many polyclonal antibodies. To be certain we are observing CAT2 specifically, we compared staining of wild‐type protoplasts with a monoclonal anti‐CAT2 specific antibody (Su *et al*., 2018) and a polyclonal anti‐catalase antibody (Fig. S2b). These showed an identical staining pattern; therefore, the more readily available polyclonal antibody was used in subsequent experiments.

**Fig. 2:**
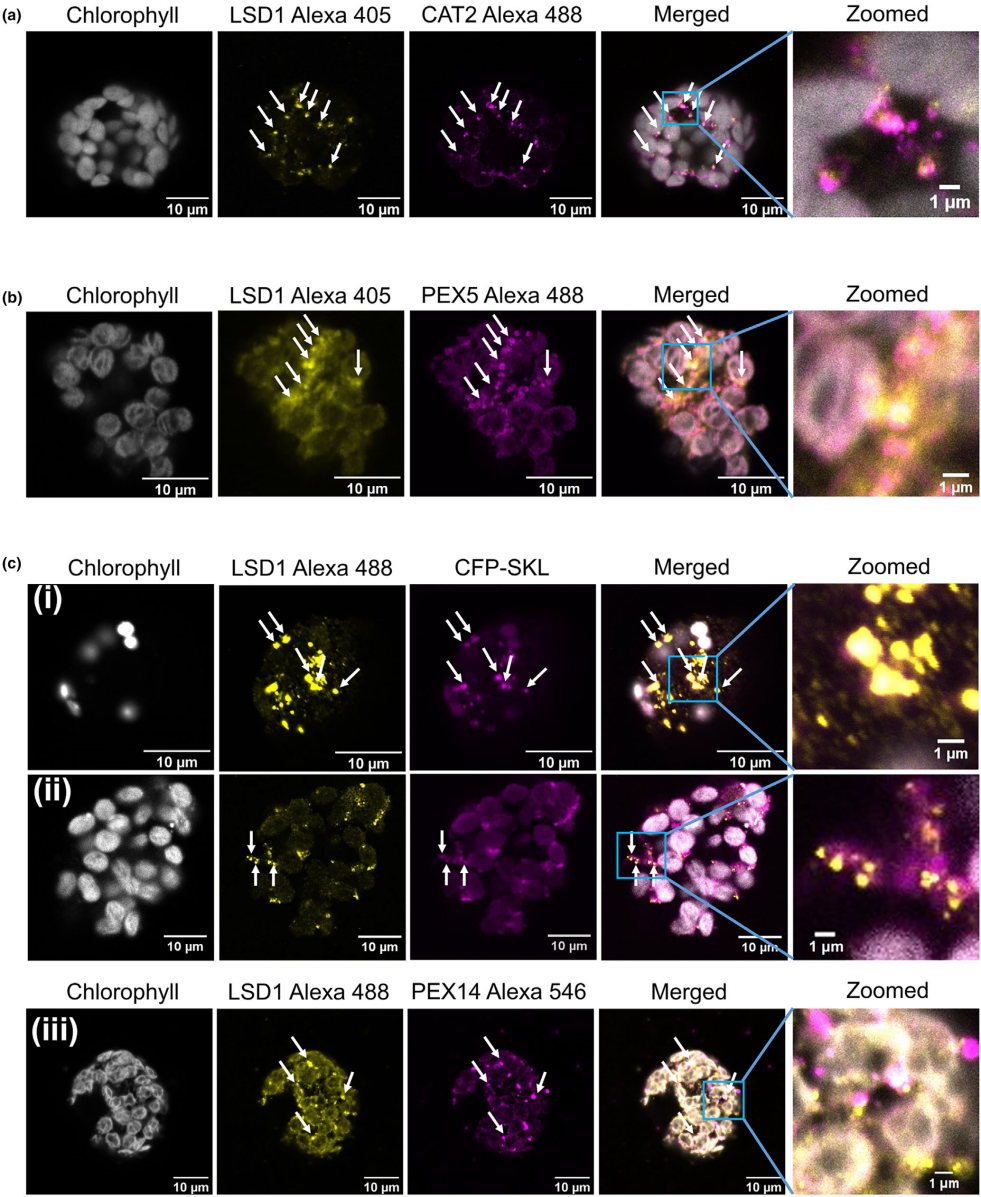
Co‐localisation of LSD1 with CAT2 or PEX5 in *Arabidopsis thaliana* protoplasts. (a) Immunofluorescent images showing the co‐localisation (white arrows) of endogenous LSD1 (yellow) and CAT2 (magenta) in protoplasts. LSD1 and CAT2 were stained with specific primary antibodies and visualised through Alexa dye‐labelled secondary antibodies (Alexa 405 for the LSD1 and Alexa 488 for the CAT2). Chl is shown in grey. Bar, 10 μm. (b) Immunofluorescent images showing the co‐localisation of endogenous LSD1 (yellow) and PEX5 (magenta) in protoplasts. Both LSD1 and PEX5 were stained with their specific antibodies and visualised through Alexa dye‐labelled secondary antibodies (Alexa 405 for the LSD1 and Alexa 488 for the PEX5). Chl is shown in grey. Bar, 10 μm. (c) LSD1 is partially localised with peroxisomes in protoplasts. (i, ii) LSD1 was stained with a specific LSD1 primary antibody and visualised through Alexa dye‐labelled secondary antibodies (Alexa 488, shown in yellow). A CFP‐tagged SKL peroxisomal marker (shown in magenta) was transformed to localise peroxisomes in protoplasts. The CFP‐SKL marker localises to small motile structures *c*. 1 μm in size, typical of peroxisomes. Chl is shown in grey. Bar, 10 μm. (iii) LSD1 was stained with a specific LSD1 primary antibody and visualised through Alexa dye‐labelled secondary antibodies (Alexa 488, shown in yellow). Peroxisomal PEX14 was stained with a specific PEX14 primary antibody and visualised through Alexa dye‐labelled secondary antibodies (Alexa 546, shown in magenta). Chl is shown in grey. Bar, 10 μm.

In protoplasts from wild‐type leaves, the endogenous cytosolic LSD1 co‐localised with the endogenous CAT2 protein in the granule‐like structures (Fig. 2a). Endogenous PEX5 appears as ring‐like structures, possibly bound to the peroxisome membrane, that partially co‐localise with LSD1 (Fig. 2b). To determine whether a portion of the LSD1 is present in peroxisomes because of the formation of a complex with CAT2 and PEX5, a peroxisomal matrix marker CFP‐SKL was transiently expressed in protoplasts, and the location of endogenous LSD1 was detected by immunofluorescence (Fig. 2ci,ii). Endogenous PEX14, a peroxisomal membrane marker, was also evaluated (Fig. 2ciii). Confocal imaging demonstrated the partial co‐localisation of endogenous LSD1 with both CFP‐SKL and PEX14 peroxisome markers.

### LSD1 undergoes phase separation *in vitro*

**Fig. 3:**
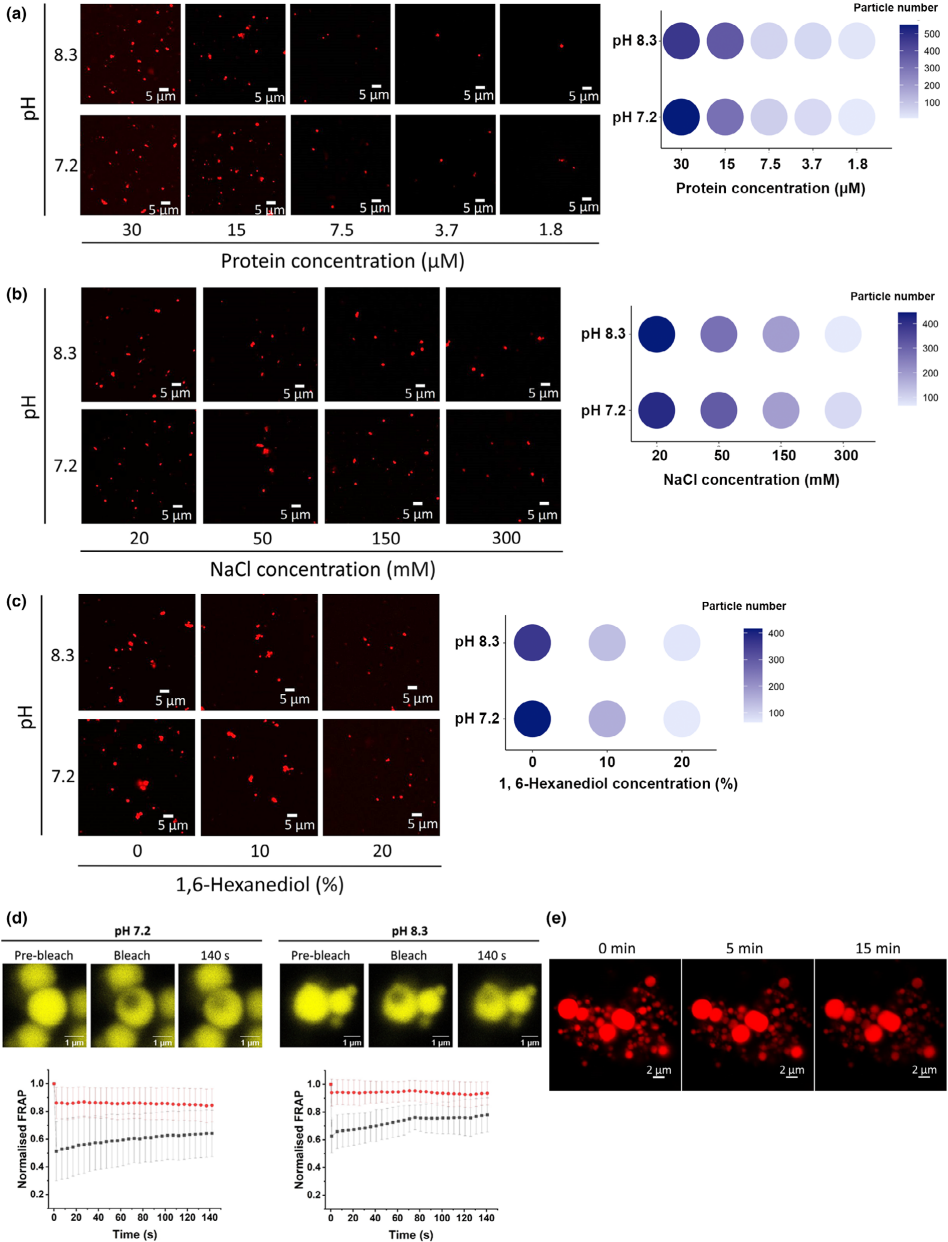
Recombinant *Arabidopsis thaliana* LSD1 undergoes phase separation *in vitro*. (a) Concentration‐dependent LSD1 condensate formation. Recombinant LSD1 was labelled with an Atto‐550 dye (shown in red) and its condensation at the indicated pH values and concentrations was observed using confocal microscopy. Bar, 5 μm. Inset: Phase diagram of LSD1 showing the number of condensates (> 0.1 μm) for each condition. Condensate numbers were calculated from a 45 177.5 μm^2^ area. (b) Effect of salt on condensate formation. Confocal images showing the formation of LSD1 condensates (shown in red) under various concentration combinations of pH and NaCl with constant protein concentration (10 μM). Bar, 5 μm. Inset: Phase diagram of LSD1 showing the number of condensates (> 0.1 μm) with the NaCl concentration ranging from 300 mM to 20 mM at two different pHs. Condensate numbers were calculated from a 45 177.5 μm^2^ area. (c) Effect of 1,6‐hexanediol on condensate formation. Representative fluorescence microscopy images showing LSD1 condensates (shown in red) under various concentration combinations of pH and 1,6‐hexanediol with constant protein concentration (10 μM). Bar, 5 μm. Inset: Phase diagram of LSD1 showing the number of condensates (> 0.1 μm) with the 1,6‐hexanediol concentration at 0% (v/v), 10% (v/v) and 20% (v/v) at two different pHs. Condensate numbers were calculated from a 45 177.5 μm^2^ area. (d) Fluorescence recovery after photobleaching (FRAP) shows the slow recovery rates of LSD1 condensates (shown in yellow) at different pH values (10 μM LSD1 protein in 20 mM HEPES pH 7.2 and 20 mM NaCl, or 20 mM HEPES pH 8.3 and 20 mM NaCl). The bleaching events occurred at the second image capture. Upper panel: represented images of LSD1 condensates at the pre‐bleach, bleach and recovery states. Lower panel: quantification of FRAP. Black: control, red: recovery after bleaching. Bar, 1 μm. Data are presented as mean ± SD of five biological repeats. (e) 10 μM of LSD1 was prepared in 20 mM NaCl at pH 8.3, and the time‐lapse microscopy showed LSD1 condensates (shown in red) in direct contact without any evidence of fusion or fission events. Bar, 2 μm.

During the preparation of recombinant proteins for MST, it was noticed that LSD1 tended to aggregate at high protein concentrations. Recombinant LSD1 protein displayed a high phase separation ability at concentrations as low as 1.8 μM and formed spherical droplets at different protein concentrations and pH values (Fig. 3a), reagents that mimic the crowding environment in the cell (Fig. S3b) and salt concentrations (Figs 3b, S3c). High salt concentrations dissolve LSD1 condensates, while pH has limited effects on the phase separation of LSD1. The addition of the crowding agent PEG550 stabilised/facilitated condensate formation, but it was not essential. The addition of 1,6‐hexanediol, which disrupts hydrophobic interactions, disrupted the LSD1 condensates with limited efficiency (Fig. 3c). Fluorescence recovery after photobleaching experiments showed that the intensity of the Fluor‐550‐labelled LSD1 fluorescence signal recovered partially at a slow rate after photobleaching (Fig. 3d). Time‐lapse imaging revealed no significant liquid‐like dynamic fusion/fission events (Fig. 3e). Together, these results demonstrate that LSD1 proteins undergo phase separation into gel‐like condensates *in vitro*.

### Zinc finger domains drive the phase separation of LSD1

LSD1 contains three zinc finger domains and one C‐terminal disordered region (Fig. S3a). The zinc finger domains are positively charged, while the disordered C‐terminal region is negatively charged (Fig. S3a), suggesting that these regions function in mediating protein–protein interactions. To pinpoint the key region(s) for phase separation, we purified the disordered region of the LSD1 sequence (residues 128–184 aa), as well as the three zinc finger domains (zinc finger 1: 1–46 aa; zinc finger 2: 47–93 aa; zinc finger 3: 94–127 aa); (Fig. S3d). Phase separation ability was tested *in vitro* using the optimal conditions for the full‐length LSD1. Surprisingly, the purified C‐terminal disordered region did not form spherical droplets *in vitro*. Rather, zinc finger domains 1 and 2 formed condensates like the full‐length LSD1 protein (Fig. S3d). However, the addition of 10 mM of H_2_O_2_ or β‐mercaptoethanol (β‐ME) did not affect the morphology of LSD1 condensates, at either pH 7.2 or pH 8.3 (Fig. S3e).

### Phase separation of LSD1 recruits CAT2 and PEX5

**Fig. 4:**
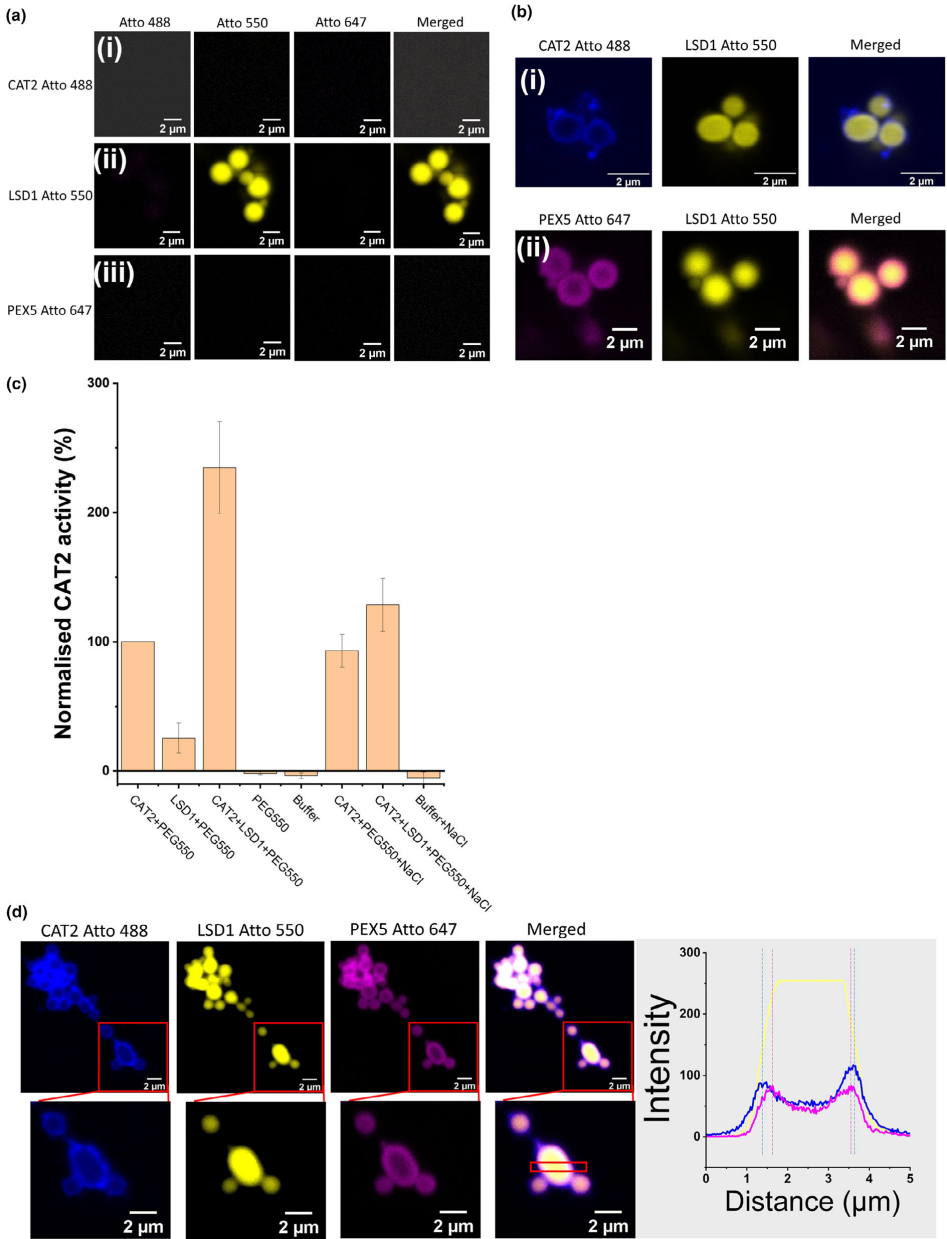
Phase separation of *Arabidopsis thaliana* LSD1 recruits PEX5 and CAT2, resulting in the upregulation of CAT2 activity *in vitro*. (a) LSD1 alone forms condensates. 10 μM of recombinant (i) CAT2, (ii) LSD1 and (iii) PEX5 were labelled with Atto 488, Atto 550 and Atto 647 dyes, respectively, and their ability to undergo phase separation as individual proteins was examined using confocal imaging. Only LSD1 could undergo phase separation and form condensates (shown in yellow), whereas PEX5 alone and CAT2 alone do not undergo phase separation. Bar, 2 μm. (b) LSD1 condensates can recruit PEX5 and CAT2 independently. 10 μM of recombinant LSD1, CAT2 and PEX5 were labelled with Atto 550 (shown in yellow), Atto 488 (shown in blue) and Atto 647 (shown in magenta), respectively. The abilities of LSD1 condensates to recruit (i) CAT2 or (ii) PEX5 were monitored using confocal imaging. The results indicated that LSD1 condensate absorbed CAT2 or PEX5 to the surfaces, but both CAT2 and PEX5 were excluded from the centre of the condensates. Bar, 2 μm. (c) LSD1 binding stimulates CAT2 activity. 10 μM of recombinant LSD1 was mixed with 10 μM of recombinant CAT2 to form the LSD1–CAT2 condensates and incubated at room temperature for 10 min. After incubation, 300 mM of NaCl was added as indicated in columns 6–8. LSD1‐CAT2 condensates and control samples, as indicated, were added to 30 mM of H_2_O_2_, and the catalase activity was measured by monitoring the change of 240 nm absorbance at room temperature. Data are presented as mean ± SD of three biological repeats (two repeats for CAT2 + PEG550 + NaCl). (d) LSD1, CAT2 and PEX5 form a ternary complex. 10 μM of recombinant LSD1, CAT2 and PEX5 were labelled with Atto 550 (shown in yellow), Atto 488 (shown in blue) and Atto 647 (shown in magenta), respectively. PEX5 and CAT2 were added to the LSD1 condensates sequentially, and the ability of LSD1 condensates to recruit both CAT2 and PEX5 was monitored using confocal imaging. The results indicated that LSD1 condensates recruit PEX5 on their surfaces first, then recruit CAT2. Importantly, these proteins form an onion‐like multilayer structure without mixing of individual proteins, as seen in the zoomed‐in images. Bar, 2 μm. Inset: quantification of fluorescent intensity of LSD1 Atto 550, CAT2 Atto 488 and PEX5 Atto 647 from the red box.

LSD1, CAT2 and PEX5 form a ternary complex in solution when the LSD1 concentration is sub‐micromolar (< 1 μM), as shown by MST analysis (Fig. 1b). Since LSD1 undergoes gel‐like phase separation when concentrations exceed 1.8 μM (Fig. 3a), the recruitment of CAT2 and PEX5 to LSD1 condensates *in vitro* was examined. All three full‐length proteins were recombinantly produced, labelled with Atto dyes and their abilities to undergo phase separation alone were examined. While LSD1 underwent phase separation without the addition of other proteins, neither CAT2 nor PEX5 formed condensates under these conditions (Fig. 4a).

Although it was not possible to measure the direct interaction between LSD1 and CAT2 in MST (Fig. 1a), CAT2 was clearly absorbed onto the LSD1 condensate surfaces (Fig. 4b, upper panel). The recruitment of CAT2 onto the LSD1 condensate surfaces increased CAT2 activity by a factor of 2.5 (Fig. 4c). This observation is consistent with the finding that catalase activity was decreased in the *lsd1* mutants and that the *lsd1* mutants are more sensitive to the catalase inhibitor 3‐amino‐1,2,4‐triazole than the wild‐type (Li *et al*., 2013). In addition, the absorption of the PEX5 protein onto the LSD1 condensate surfaces was observed (Fig. 4b, lower panel). These results are consistent with the suggestion that LSD1 could function as a scaffold protein (Czarnocka *et al*., 2017) by virtue of its phase separation ability.

To investigate how the phase separation of LSD1 affects the formation of a ternary complex, PEX5 and CAT2 were sequentially added to LSD1 condensates, and the formation of the ternary complex was observed using confocal imaging. Multilayer, onion‐like structures of the ternary complex were observed (Fig. 4d). The LSD1 condensates formed the core of the structure, with the PEX5 proteins outside the LSD1 core. These structures absorbed CAT2 onto the outer layer without mixing of the individual proteins.

### Redox‐dependent regulation of LSD1–PEX5–CAT2 phase separation

**Fig. 5:**
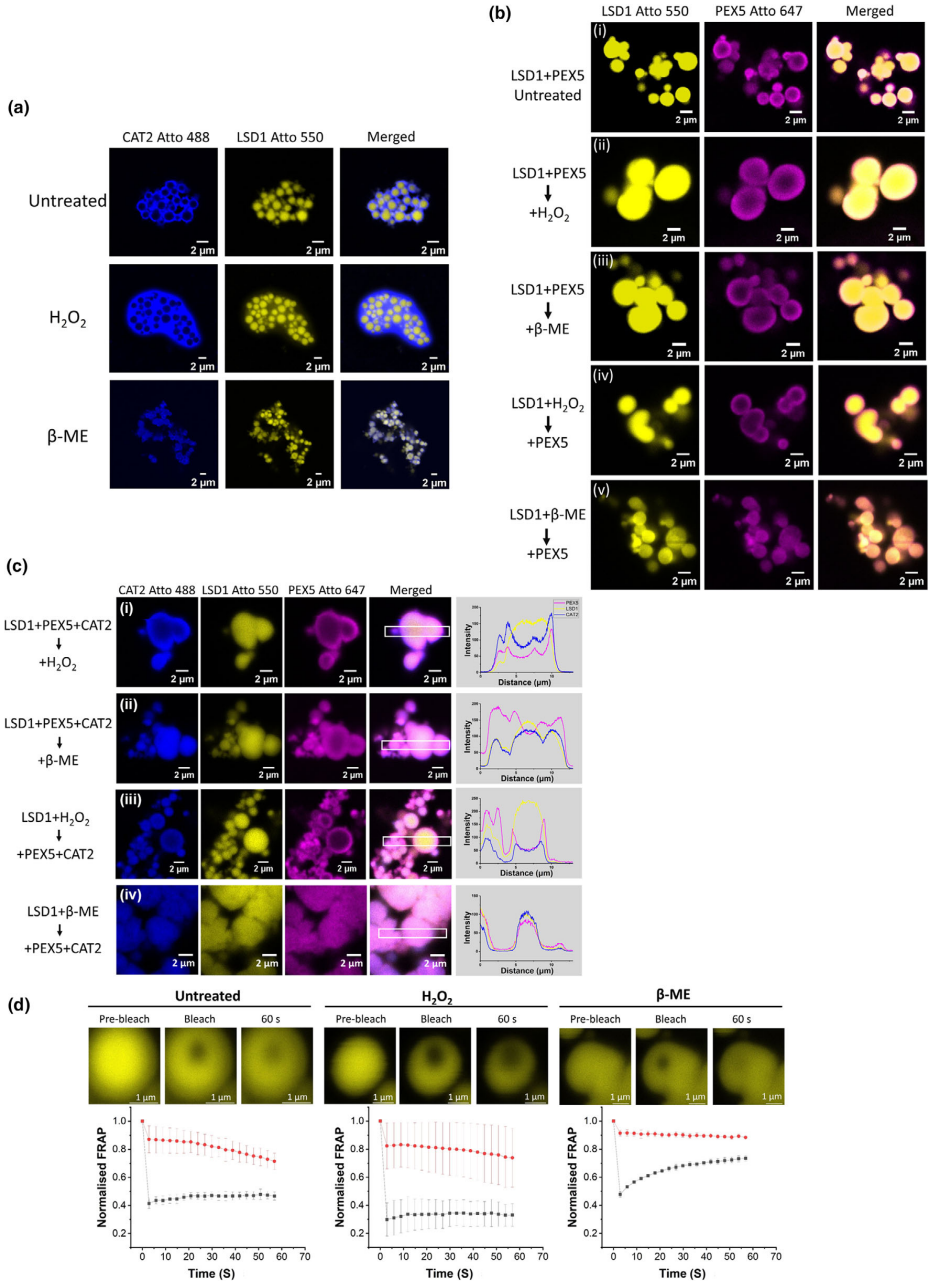
Recruitment of *Arabidopsis thaliana* PEX5 and CAT2 to LSD1 condensates is regulated by redox states. (a) LSD1 condensates (shown in yellow) recruit CAT2 (shown in blue) in a redox‐dependent manner. CAT2 was recruited to the surfaces of LSD1 condensates. The oxidised condition (10 mM H_2_O_2_, middle panel) promotes the recruitment of CAT2 to the surfaces of LSD1 condensates. However, the reduced condition (10 mM β‐ME, lower panel) causes the partitioning of CAT2 into LSD1 condensates. Bar, 2 μm. (b) The formation of the LSD1 (shown in yellow)–PEX5 (shown in magenta) condensates protects the condensates from redox treatments. Treating the pre‐formed LSD1–PEX5 condensates (i) with the oxidised condition (10 mM H_2_O_2_, (ii)) or the reduced condition (10 mM β‐ME, (iii)) does not affect the morphology of LSD1–PEX5 condensates. 10 mM H_2_O_2_ treatment on LSD1 condensates alone before the formation of LSD1–PEX5 (iv) condensates also has no effect on the morphology of LSD1–PEX5 condensates. However, 10 mM β‐ME treatment on LSD1 condensates alone before the formation of LSD1–PEX5 condensates resulted in the diffusion of PEX5 protein within the LSD1 condensate (v). Bar, 2 μm. (c) Effects of redox treatments on the LSD1 (shown in yellow)–PEX5 (shown in magenta)–CAT2 (shown in blue) condensates: (i) treating the preformed formation of LSD1–PEX5–CAT2 condensates with 10 mM H_2_O_2_; (ii) treating the preformed formation of LSD1–PEX5–CAT2 condensates with 10 mM β‐ME; (iii) treating LSD1 with 10 mM H_2_O_2_ before the addition of PEX5 and CAT2; (iv) treating LSD1 with 10 mM β‐ME before the addition of PEX5 and CAT2. Inset: quantification of fluorescent intensity of LSD1 Atto 550, CAT2 Atto 488 and PEX5 Atto 647 from the white box. Bar, 2 μm. (d) Fluorescence recovery after photobleaching (FRAP) shows that the reducing environment increases the liquid‐like property of LSD1 condensates (shown in yellow). LSD1 condensates were untreated or treated with 10 mM H_2_O_2_/β‐ME for 10 min followed by FRAP analysis within 60 s. The bleaching events occurred at the second image capture. Upper panel: represented images of LSD1 condensates at the pre‐bleach, bleach and recovery states. Lower panel: quantification of FRAP. Data are presented as mean ± SD of three biological repeats. Red: control, Black: recovery after bleaching. Bar, 1 μm.

While the addition of oxidants or reductants had no effect on the LSD1 condensates (Fig. S3e), it had a significant effect on the preformed LSD1–CAT2 condensates. The addition of 10 mM of H_2_O_2_ greatly enhanced the accumulation of CAT2 onto the LSD1 condensate surfaces (Fig. 5a, middle panel). In striking contrast, the addition of 10 mM β‐ME resulted in CAT2 penetration of the LSD1 condensates (Fig. 5a, lower panel). However, the addition of 10 mM of H_2_O_2_ or β‐ME to the preformed LSD1–PEX5 condensates did not affect their morphology (Fig. 5bi,ii,iii); PEX5 was observed on the outer layer of these complexes, as was the case in the untreated samples. Treating the LSD1 condensates with 10 mM of H_2_O_2_ before the addition of PEX5 also had no effect on the morphology of LSD1–PEX5 condensates (Fig. 5biv). Intriguingly, treating the LSD1 condensates with 10 mM β‐ME before adding PEX5 resulted in the mixture of the LSD1 and PEX5 proteins within the complex (Fig. 5bv).

Next, we examined the effects of redox treatments (10 mM H_2_O_2_ or 10 mM β‐ME) on the preformed LSD1–PEX5–CAT2 ternary complex, and the morphology and distribution of the individual proteins were analysed. Instead of forming the three‐layer structure (Fig. 4d), treatment of the preformed LSD1–PEX5–CAT2 condensates with H_2_O_2_ resulted in a mixture of the PEX5 and CAT2 proteins on the LSD1 condensate surface (Fig. 5ci). The β‐ME treatment on the preformed complex (Fig. 5cii) caused CAT2 to mix with the LSD1 in the condensate cores (Fig. 5a lower panel). However, PEX5 largely remained on the condensate surface (Fig. 5biii).

In further experiments, LSD1 condensates were treated with 10 mM H_2_O_2_ or β‐ME before the addition of PEX5 and CAT2 proteins. Exposing LSD1 condensates to an oxidised environment did not result in mixing of the PEX5 and CAT2 proteins within the LSD1 condensates. However, the arrangement of the PEX5 and CAT2 proteins within the LSD1 condensates changed. Instead of PEX5 coating the LSD1 condensate, and a CAT2 protein coat around the PEX5 proteins in the absence of redox treatments (Fig. 4d), the oxidation of LSD1 prior to the addition of PEX5 and CAT2 resulted in an exchange of PEX5 and CAT2 distributions (Fig. 5ciii). Finally, exposing LSD1 condensates to a reducing environment before ternary condensate formation resulted in a mixture of CAT2 and LSD1 proteins within the LSD1 condensates (Fig. 5civ). These results are summarised in Fig. S4(a). FRAP assays were performed to determine whether the redox environment might change the fluidity of the LSD1 condensates and therefore the distribution of PEX5 and CAT2. The results indicated that a reducing environment significantly increases the average fluorescent recovery rate from 5.2% (untreated) to 25.7% within 60 s (Fig. 5d). By contrast, an oxidising environment did not influence the fluidity of LSD1 condensates, as the recovery rate remained at 3.2%.

### Redox‐dependent regulation of the subcellular localisation of CAT2

**Fig. 6:**
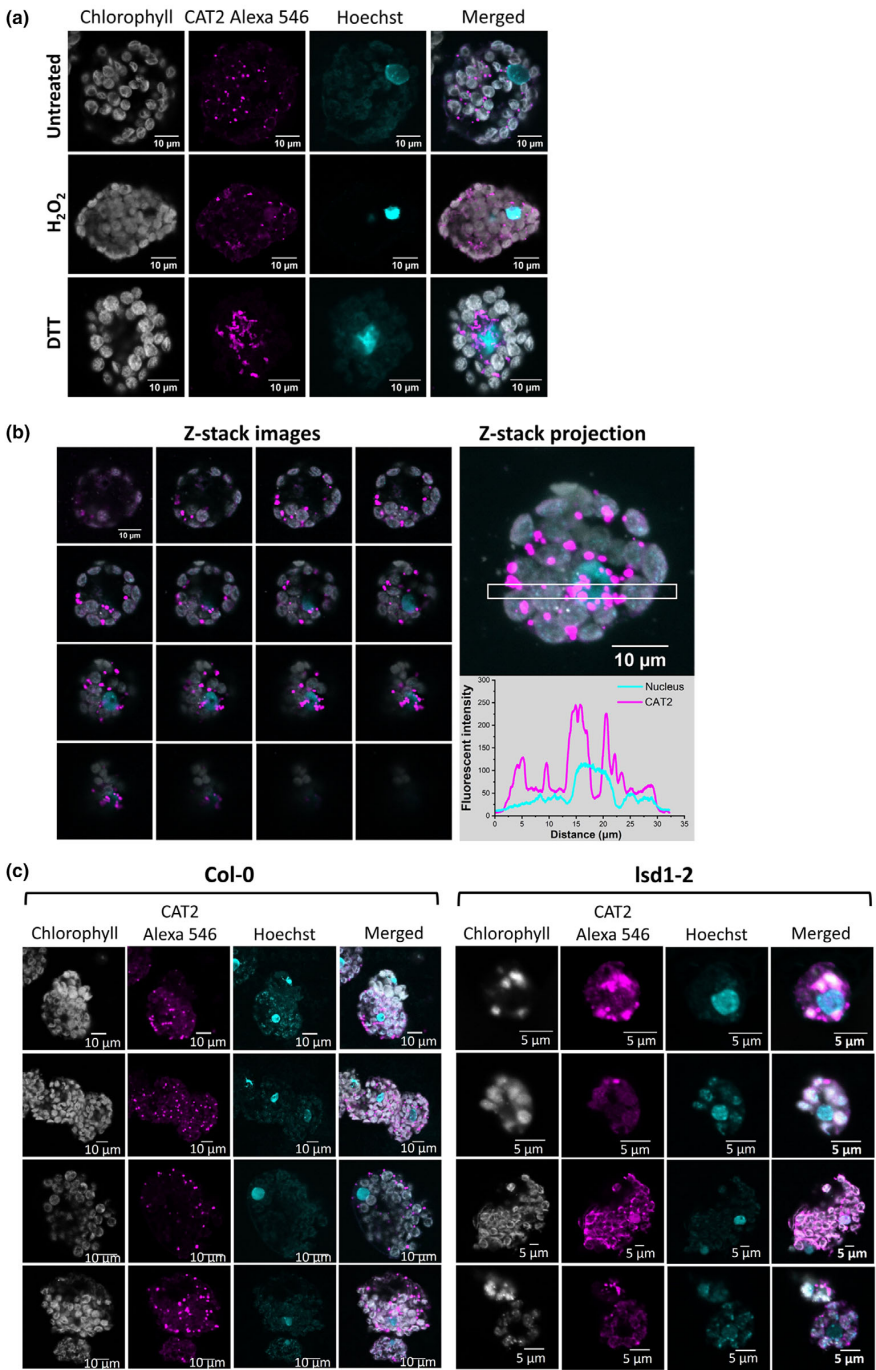
*Arabidopsis thaliana* LSD1 is required for the subcellular organisation and distribution of CAT2. (a) Redox regulates CAT2 distribution in protoplasts. Under untreated conditions, protoplasts were isolated and endogenous CAT2 was stained with an antibody captured with Alexa 546‐labelled secondary antibody (shown in magenta). Chl is shown in grey, and nuclei were stained with Hoechst (shown in cyan). CAT2 localises in the cytoplasm, presumably in the peroxisomes. DTT treatment (10 mM for 6 h at room temperature) causes CAT2 to translocate to the nucleus, while H_2_O_2_ treatment (10 mM for 6 h at room temperature) results in the diffuse localisation of CAT2 both in the cytosol and in the nucleus. Bar, 10 μm. (b) Z‐stack images showing the localisation of CAT2 in the nucleus upon DTT treatment. Sixteen images were captured with an interval of 1 μm. The Z‐stack projection image showed the overall distribution of CAT2 in the cytosol and the nucleus. The white‐boxed area was used for the fluorescent density analysis; magenta: CAT2 fluorescent signal, cyan: nuclear staining fluorescent signal. Bar, 10 μm. (c) The presence of LSD1 controls the subcellular localisation of CAT2. Left panel: The *lsd1–2* mutant results in an abnormal localisation of CAT2. The sizes of protoplasts are also smaller than those of wild‐type protoplasts. Right panel: CAT2 is restricted in defined areas, presumably in the peroxisomes. Immunofluorescent images for both *lsd1–2* and wild‐type protoplasts show the location of endogenous CAT2 (magenta), Chl (grey) and Hoechst nucleic acid stain (cyan). Four independent images are presented for both *lsd1–2* (Bar, 5 μm) and col‐0 protoplasts. Bar, 10 μm.

Next, we explored the effects of the redox environment on the subcellular localisation of the CAT2 protein. As shown earlier, CAT2 was localised in the cytoplasm, presumably in the peroxisomes and/or granule‐like structures, and those structures were distributed evenly throughout the cell (Fig. 6a, upper panel). The H_2_O_2_ treatment caused CAT2 staining to be more diffuse in the cytosol (Fig. 6a, middle panel). Under reducing conditions, however, CAT2‐containing structures were more concentrated around the nuclei. Moreover, some of the CAT2‐containing structures were co‐localised with nuclei (Fig. 6a, lower panel). The intracellular distribution of CAT2 was further analysed using confocal Z‐stack images, which revealed that a proportion of CAT2 was internalized in the nucleus (Fig. 6b). As a control, the effect of an altered redox environment on the intracellular distribution of a different peroxisomal protein, glycolate oxidase, was examined (Fig. S4b). While H_2_O_2_ treatment resulted in a more diffuse staining of glycolate oxidase, reducing conditions had no effect on the localisation of glycolate oxidase. Therefore, the redox‐dependent intracellular localisation of CAT2 is a specific property of this protein.

### LSD1 controls CAT2 cellular distribution in Arabidopsis

Having demonstrated the interactions between LSD1 and CAT2 *in vitro* (Figs 1, 3, 4, 5) and the co‐localisation of LSD1 and CAT2 in protoplasts (Fig. 2), we explored the biological importance of LSD1 condensates on CAT2 localisation. The effects of the loss of LSD1 proteins on CAT2 distribution were evaluated in *lsd1–2* mutants that have an undetectable presence of LSD1 using immunofluorescence (IF) (Fig. S23aiii,iv). The *lsd1‐2* mutants have much smaller mesophyll protoplasts than the wild‐type (Czarnocka *et al*., 2017). A punctate pattern of CAT2 staining was observed in the wild‐type, whereas CAT2 distribution was changed in the mutants, appearing more diffuse (Fig. 6c), suggesting a role for LSD1 in CAT2 localisation *in vivo*.

## Discussion

In this study, we demonstrate that CAT2 forms a phase separated structure with LSD1 that not only greatly enhances its activity but also increases its localisation within the nucleus. We present evidence that LSD1 recruits CAT2, and the peroxisome import receptor PEX5, into a ternary complex that has LSD1 at the core, with PEX5 on the surface and CAT2 bound to the PEX5 in a layered structure (Fig. 4d). The properties of these complexes are modified in response to changes in the redox environment, which has differential effects on the distribution of the individual proteins within the complex (Fig. S5a).

Zinc fingers 1 and 2 of LSD1 can undergo condensate formation and are also required for interactions with other proteins (Kaminaka *et al*., 2006; Coll *et al*., 2010; He *et al*., 2011; Li *et al*., 2013). The redox states of cysteines have been demonstrated to have significant impacts on condensate formation both in plants (X. Huang *et al*., 2021) and in animals (Ambadipudi *et al*., 2017; La Cunza *et al*., 2021). Zinc fingers in proteins can act as redox switches (Ilbert *et al*., 2006), and the zinc fingers in LSD1 are sensitive to changes in hydrogen peroxide concentration (Dietrich *et al*., 1997). Active LSD1 protein is converted into an inactive form by redox signals generated by modulation of the plastoquinone pool in the thylakoid membranes, and addition of Dithiothreitol (DTT) converts LSD1 dimers to monomers (Chai *et al*., 2015). Reduction of disulphide bonds by β‐ME could explain the increased fluidity observed within the condensates. Although the LSD1 condensates do not show an increase in fluidity (as measured by FRAP) upon the addition of H_2_O_2_, the addition of H_2_O_2_ to preformed LSD1–CAT2 complexes massively increased CAT2 recruitment onto the condensate surface. These properties could explain the previous observation that the LSD1 interactome is highly sensitive to redox conditions (Czarnocka *et al*., 2017).

The CAT2 and LSD1 proteins colocalise in protoplasts. In addition, LSD1 partially colocalises with PEX5, a peroxisomal matrix marker CFP‐SKL, and the peroxisomal membrane marker PEX14 (Fig. 2) suggesting the possibility that a portion of LSD1 is localised to peroxisomes via its interactions with PEX5. The significance of a peroxisome localised pool of LSD1 is unclear but could function to increase catalase activity in that compartment. Additionally, under the redox conditions pertaining in protoplasts, which are highly oxidised compared to seedlings (C. Lett & C. Foyer in preparation), recruitment of PEX5 to LSD1 condensates could then recruit other PEX5 targeted peroxisomal proteins, thereby reducing import into peroxisomes in a redox sensitive manner. Under oxidising conditions, the import of proteins into mammalian peroxisomes is decreased via two mechanisms: inhibition of PEX5 recycling (Apanasets *et al*., 2014) and phosphorylation of ser232 in PEX14 (Okumoto *et al*., 2020). While it has not been shown that similar mechanisms operate in plants, the relevant amino acid residues are conserved, and our *in vivo* data (Figs 6a, S4b) provide evidence for decreased peroxisomal import *in vivo* in H_2_O_2_‐treated protoplasts. Conversely, NADPH specifically inhibited import of proteins into glyoxysomes (Pool *et al*., 1998). Peroxisomal import may, therefore, depend on a specific window of redox state, regulated at multiple levels. Interestingly, inhibition of peroxisome protein import in *C. elegans* through downregulation of PEX5 led to peroxisome retrograde signalling, which upregulated catalase expression (Rackles *et al*., 2021).

**Fig. 7:**
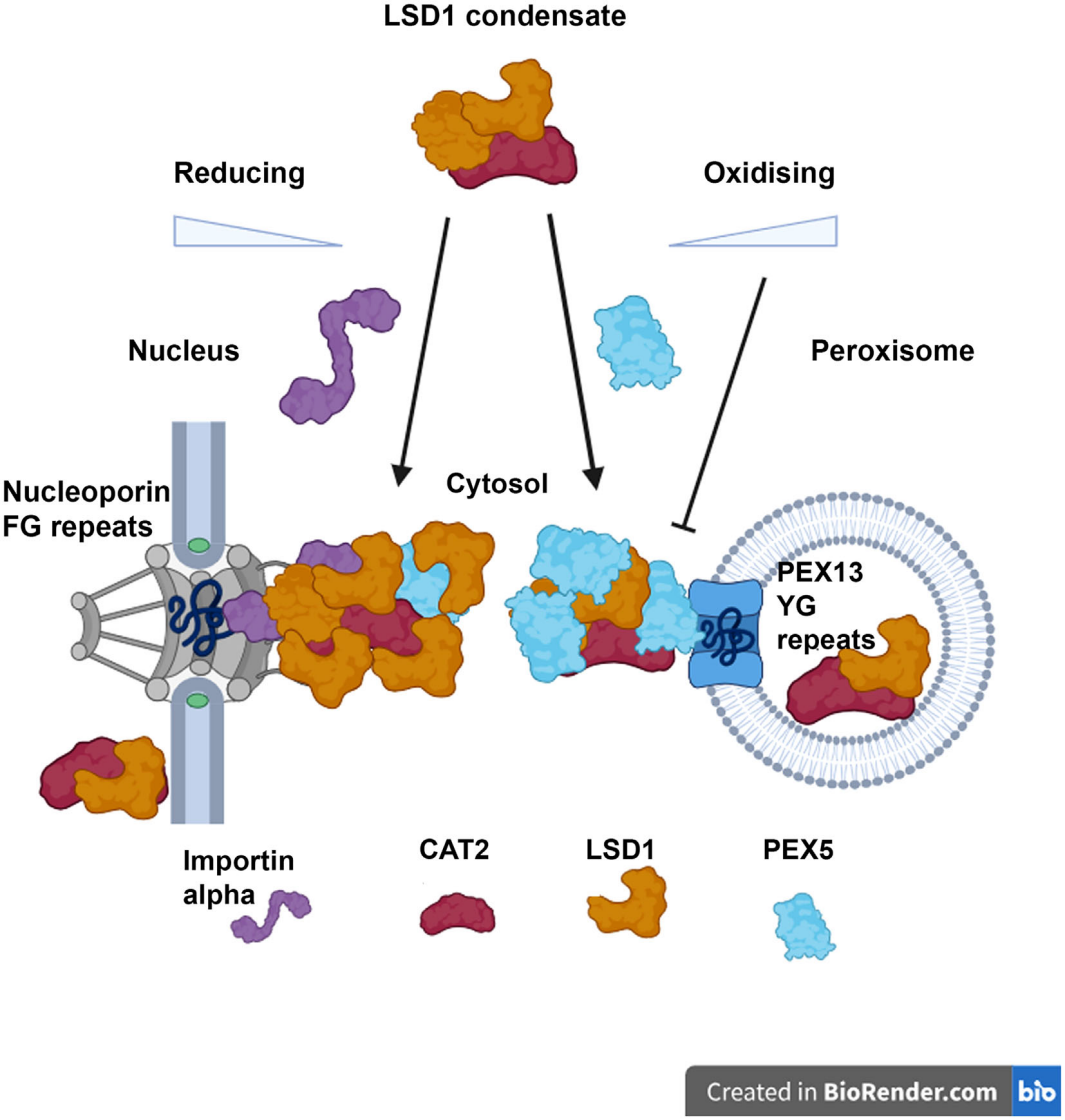
Model for redox‐regulated targeting of *Arabidopsis thaliana* CAT2 between the nucleus and peroxisomes. CAT2 and PEX5 bind to LSD1 condensates in the cytosol. Under reducing conditions, LSD1 is more accessible to bind the nuclear transport receptor importin α (left hand arrow), LSD1 and some CAT2 are targeted to the nucleus. Under more oxidising conditions, PEX5 is more accessible on the surface of condensates (right hand arrow) and promotes CAT2 targeting to peroxisomes, along with some LSD1, which upregulates catalase activity. Under higher levels of oxidation, peroxisome import is blocked (illustrated by a blunt arrow), retaining CAT2 and LSD1 in the cytosol. Not to scale and no stoichiometry is implied in this schematic diagram. Importin alpha: purple, CAT2: red, LSD1: yellow and PEX5: light blue. Created in BioRender. Baker (2025) https://BioRender.com/bnk67r3.

We demonstrate that LSD1 is required for proper CAT2 localisation (Fig. 6c). LSD1 contains a nuclear targeting signal and interacts with the nuclear transport receptor importin α (He *et al*., 2011). Intriguingly, the importin α binding site of LSD1 also lies within the Zn fingers. We present a model (Fig. 7) of how the redox environment regulates condensate properties and could influence the targeting of catalase between the peroxisomes, cytosol and nucleus. We speculate that the effect of reducing conditions, which promote the internalisation of CAT2 and PEX5 within the condensates, increases accessibility of LSD1 for interaction with importin α and hence nuclear transport of condensates (Figs 7, S4a). By contrast, under oxidising conditions, LSD1 is coated by CAT2 and PEX5, reducing its accessibility (Fig. S4a). Import of proteins into the nucleus has long been known to occur through a gel‐like phase in the lumen of the nuclear pore. Nuclear transport receptors can penetrate this phase and bring their cargo into the nucleus before being recycled (Ng *et al*., 2021; Wing *et al*., 2022). More recently, peroxisomal import was also shown to occur via a phase‐separated mechanism. The PEX13 component of the translocation machinery has a YG‐rich domain which can a form a phase‐separated state into which cargo‐bound PEX5 can diffuse (Gao *et al*., 2022; Ravindran *et al*., 2023) and mutations which impact this property also inhibit PTS1 import (Gao *et al*., 2022, Ravindran *et al*., 2023).

What is the broader significance of these findings? The importance of catalase in stress signalling is well established, and catalases are targets of many pathogen effectors of bacteria, viruses and oomycetes, which manipulate their transcription (Zhu *et al*., 2023), activity (Mathioudakis *et al*., 2013; Roshan *et al*., 2018), stability (Zhang *et al*., 2015; You *et al*., 2022) and location (Zhang *et al*., 2015). Root knot nematodes even produce an effector with catalase activity to manipulate cell survival (Zhu *et al*., 2024). We propose that the relocation of catalase to the nucleus is a means to provide antioxidant capacity, specifically the ability to remove hydrogen peroxide generated by nuclear‐localised superoxide dismutase under stress conditions (Karpinska & Foyer, 2024). The mechanism of activation and recruitment of catalase to the nucleus by LSD1 biomolecular condensates may provide protection for LSD1 (and other zinc finger transcription factors) against hydrogen peroxide oxidation of Zn finger domains. Additionally, the ability to precisely regulate nuclear antioxidant capacity is important to protect against DNA damage and for redox regulated gene transcription (Zavaliev *et al*., 2020). This latter is a key mechanism for plants to respond to biotic and abiotic stress.

## Competing interests

None declared.

## Author contributions

All authors contributed to the design of the work. AB, CHF and MW obtained funding. C‐CL performed all experiments. All authors contributed to the analysis and interpretation of results. AB and C‐CL wrote the first draft of the manuscript. CHF and MW revised the manuscript, and all authors approved the final manuscript.

## Disclaimer

The New Phytologist Foundation remains neutral with regard to jurisdictional claims in maps and in any institutional affiliations.

## Supporting information


**Fig. S1** Production of recombinant *Arabidopsis thaliana* proteins used in this study.
**Fig. S2** Cellular expression and distribution of endogenous *Arabidopsis thaliana* LSD1.
**Fig. S3** Recombinant *Arabidopsis thaliana* LSD1 undergoes phase separation *in vitro*.
**Fig. S4** Effect of redox treatment on the localisation of *Arabidopsis thaliana* glycolate oxidase in protoplasts.
Please note: Wiley is not responsible for the content or functionality of any Supporting Information supplied by the authors. Any queries (other than missing material) should be directed to the *New Phytologist* Central Office.

## Data Availability

The data that support the findings of this study are available in the manuscript and the Supporting Information of this article (Figs S1–S4).
