# Supplementary material for: Redox regulation of LSD1/CATALASE 2 phase separation condensates controls location and functions
Source: New Phytol. 2025 Jul 21;247(6):2824–38. doi: 10.1111/nph.70374 (PMC12371180; doi:10.1111/nph.70374)

## *New Phytologist* Supporting Information

Article title: Redox regulation of LSD1/CATALASE 2 phase separation condensates controls location and functions

Authors: Chi-Chuan Lin, Christine H. Foyer, Megan Wright, and Alison Baker

Article acceptance date: 19 June 2025

The following Supporting Information is available for this article:

**Fig. S1** **Production of recombinant *Arabidopsis thaliana* proteins used in this study.**

Supplementary 1A: SDS-PAGE demonstrating integrity and purity of recombinant CAT2, PEX5, and LSD1 purified from *E.coli*. CAT2: theoretical molecular weight (including the 6xHis-tag): 59.3 kDa, observed molecular weight: 65 kDa. PEX5: theoretical molecular weight (including 6xHis-tag): 83.3 kDa, observed molecular weight: 80 kDa. LSD1: theoretical molecular weight (including the 6xHis-tag): 22 kDa, observed molecular weight: 27 kDa.

kDa: kilodalton.

**
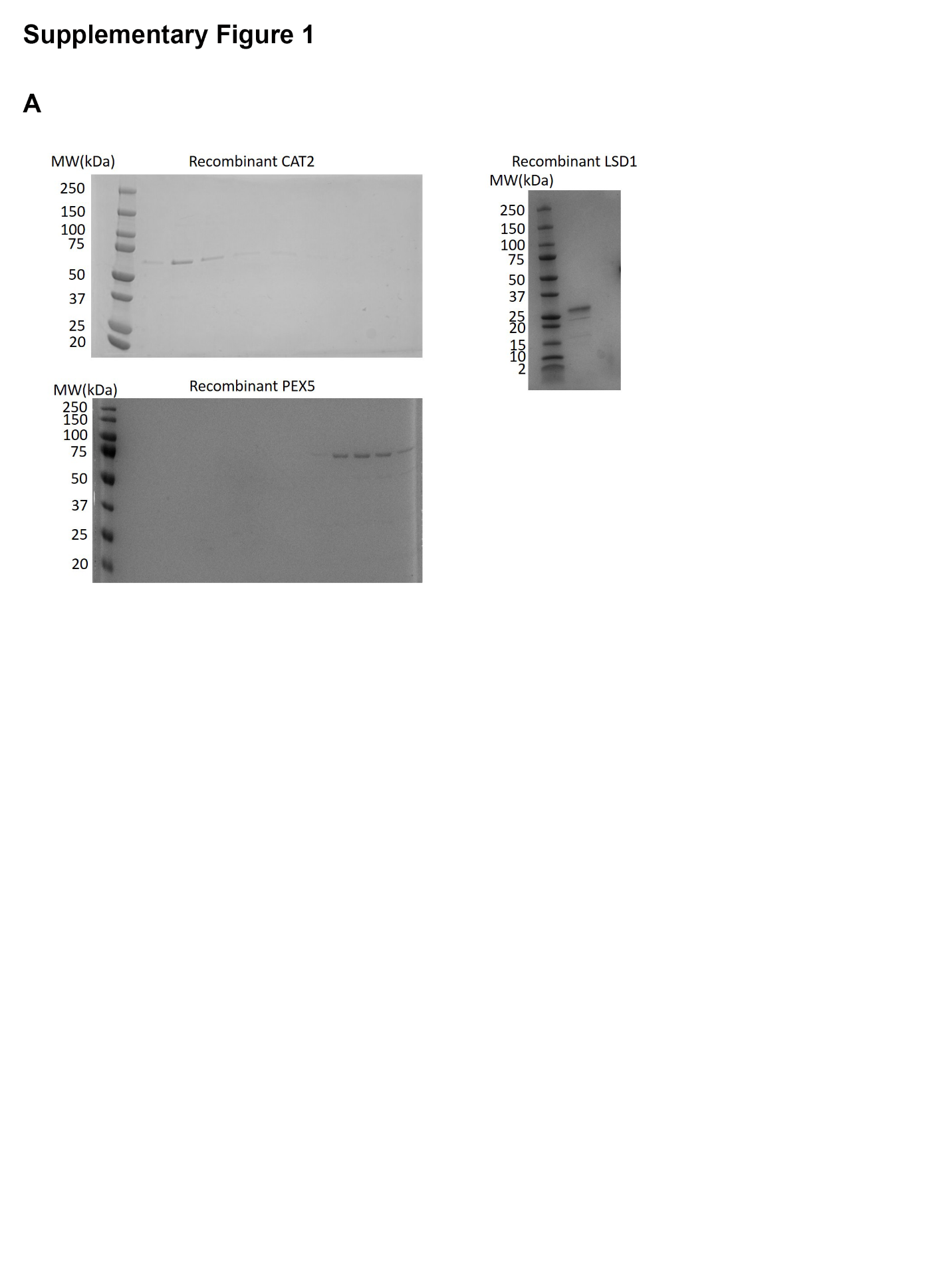
**

**Fig. S2** **Cellular expression and distribution of endogenous *Arabidopsis thaliana* LSD1.**

Supplementary 2A: Immunofluorescence staining using an anti-LSD1 antibody (shown in yellow) was used to examine the expression and distribution of endogenous LSD1 in protoplasts. (i) detecting LSD1 in wild type protoplast nucleus. (ii) detecting LSD1 in wild type protoplast cytoplasm. (iii) demonstrating absence of LSD1 in protoplast from *lsd1-2* mutant. (iv) control image using secondary antibody alone in *lsd1-2* mutant protoplast. chlorophyll is shown in grey. Scale bar=10 µm.

Supplementary 2B: Immunofluorescence staining using both polyclonal (magenta) and monoclonal (yellow) anti-CAT2 antibodies in protoplasts to compare their specificity against CAT2. Chlorophyll is shown in grey, and nuclei are shown in cyan. Three protoplasts were shown. Scale bar=10 µm.

µm: micrometer.


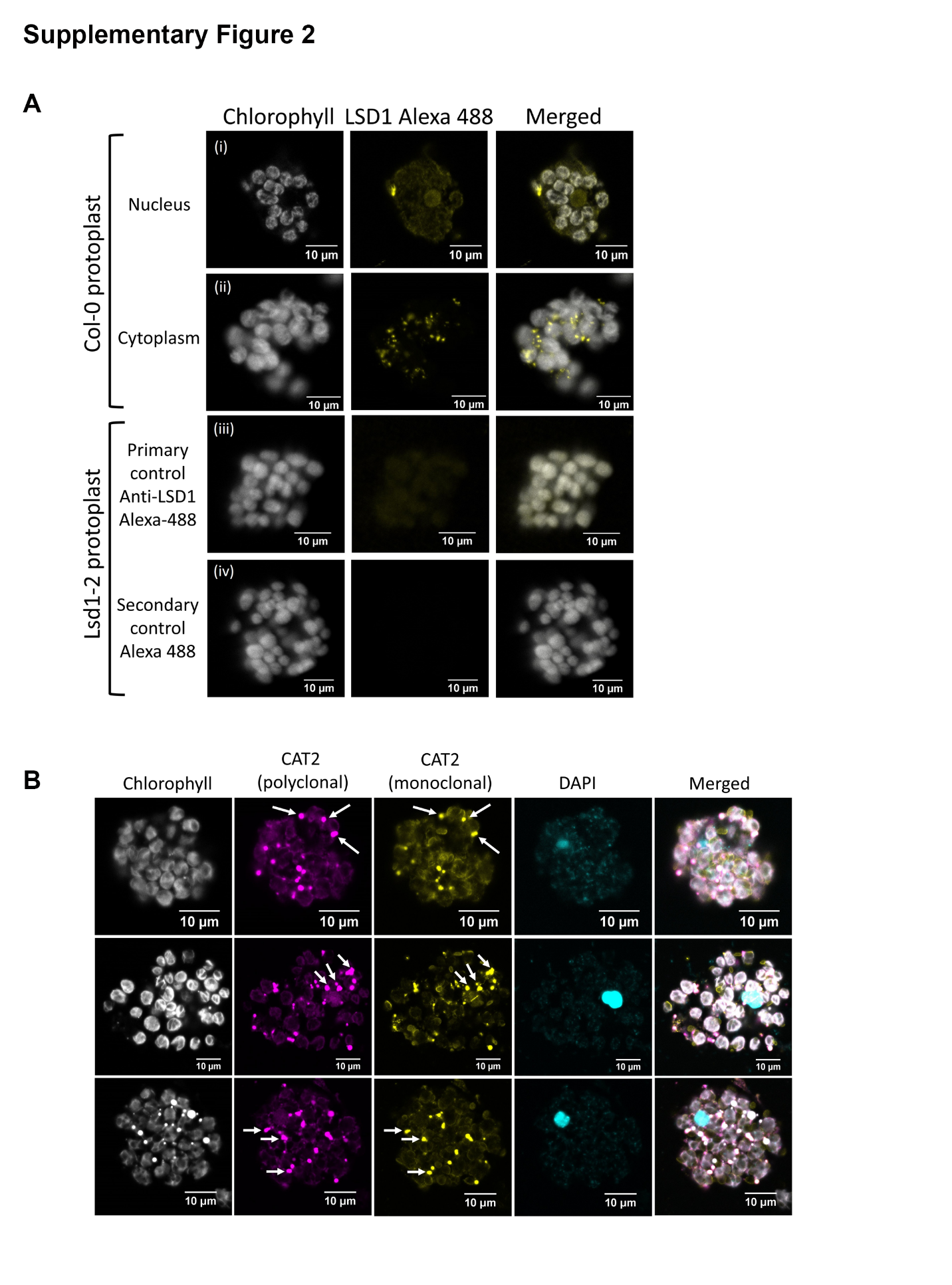


**Fig. S3** **Recombinant *Arabidopsis thaliana* LSD1 undergoes phase separation *in vitro*.**

Supplementary 3A: Sequence properties of LSD1 protein. The net charge per residue (NCPR) distribution of LSD1 revealed that the C-terminal region is negatively charged while the Zinc-finger regions are positively charged. The disordered regions of LSD1 were predicted using PONDR.

Supplementary 3B: Representative images of 10 μM recombinant LSD1 condensate (shown in red) formation in the absence and presence of 10% (w/v) PEG550. Scale bar=10 μm.

Supplementary 3C: Confocal images showing the formation of LSD1 condensates (shown in red) with various concentration of KCl at 2 different pHs with constant protein concentration (10 μM) and NaCl concentration (20 mM).  Scale bar=5 μm.

Supplementary 3D: Schematic diagrams of LSD1 regions used in this study. Individual regions were cloned, and recombinant proteins were produced for the phase separation assay (shown in red) to identify the critical region(s). Scale bar=5 μm.  Inset: SDS-PAGE for recombinant LSD1 individual domains, Zinc-1: theoretical molecular weight (including the 6xHis-tag): 7.4 kDa, observed molecular weight: 10 kDa. Zinc-2: theoretical molecular weight (including the 6xHis-tag): 7.2 kDa, observed molecular weight: 12 kDa. Zinc-3: theoretical molecular weight (including the 6xHis-tag): 6.0 kDa, observed molecular weight: 12 kDa. Disordered region: theoretical molecular weight (including the 6xHis-tag): 8.2 kDa, observed molecular weight: 14 kDa.

Supplementary 3E: The morphology of LSD1 condensates (shown in red) were examined at two different pH values upon redox treatments. 10 mM β-ME or 10 mM H_2_O_2_ was added to the LSD1 condensates and incubated for 10 minutes before confocal imaging analysis. Scale bar=10 μm.

μm: micrometer. μM: micromolar. mM: minimolar. kDa: kilodalton.


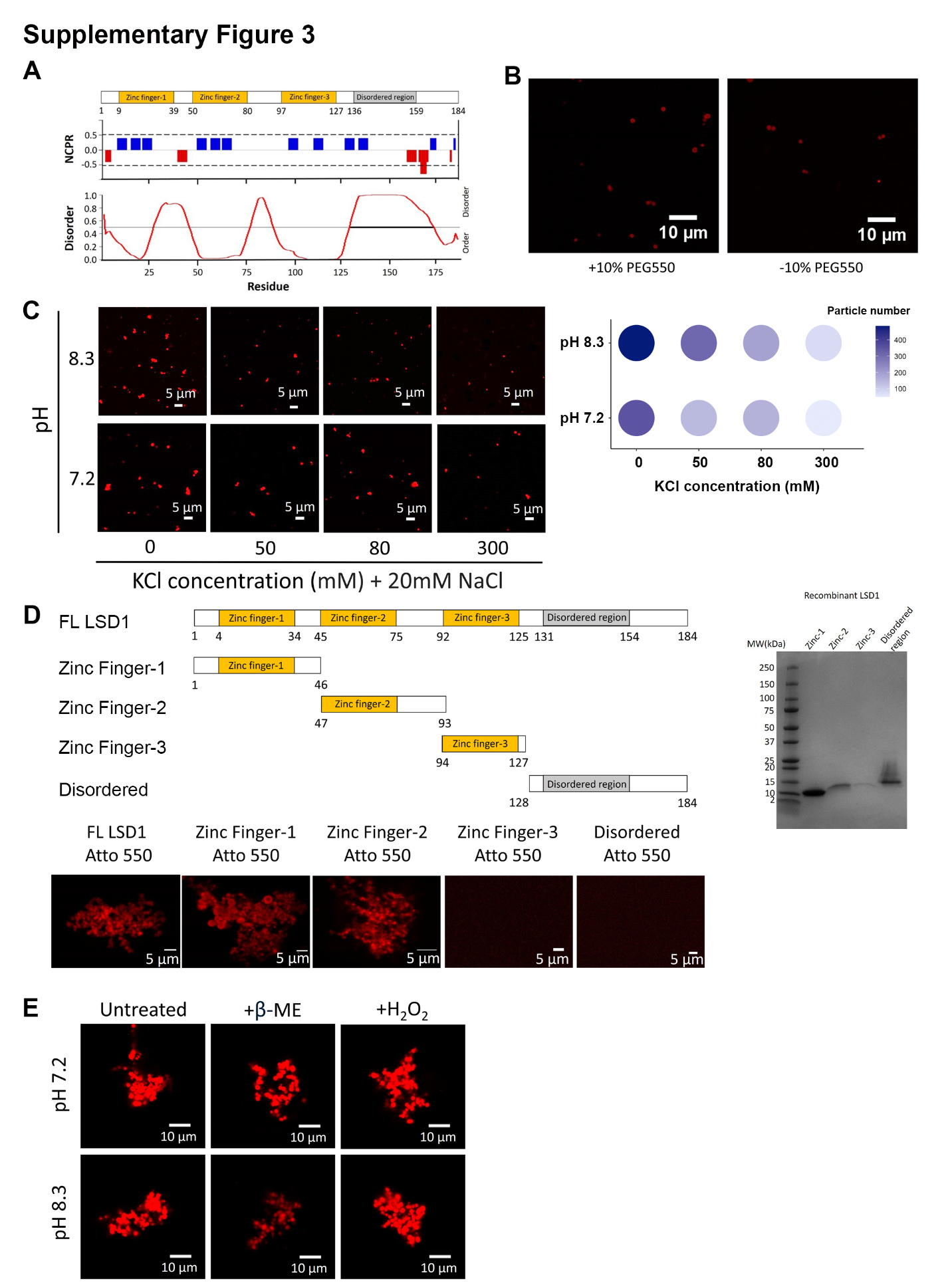


**Fig. S4** **Effect of redox treatment in the localisation of *Arabidopsis thaliana* glycolate oxidase in protoplasts.**

Supplementary 4A: Summary of in vitro LSD1-PEX5-CAT2 condensate formation under different redox treatment. LSD1 is shown in yellow, PEX5 is shown in blue, and CAT2 is shown in magenta. The corresponding figures for each step was indicated.

Supplementary 4B: Redox treatment does not affect peroxisomal glycolate oxidase distribution in protoplasts. Under untreated condition, glycolate oxidase localises in the cytoplasm, presumably in the peroxisomes. Different from CAT2, DTT treatment (10 mM for 6 hours at room temperature) does not cause glycolate oxidase to translocate to the nucleus. However, H_2_O_2_ treatment (10 mM for 6 hours at room temperature) also results in the diffused localisation of glycolate oxidase both in cytosol and nucleus. Protoplasts were isolated and endogenous peroxisomal glycolate oxidase was stained with antibody captured with Alexa 546-labelled secondary antibody (shown in magenta). Chlorophyll is shown in grey, and nuclei were stained with Hoechst (shown in cyan). Scale bar=10 μm.

μm: micrometer. mM: minimolar.


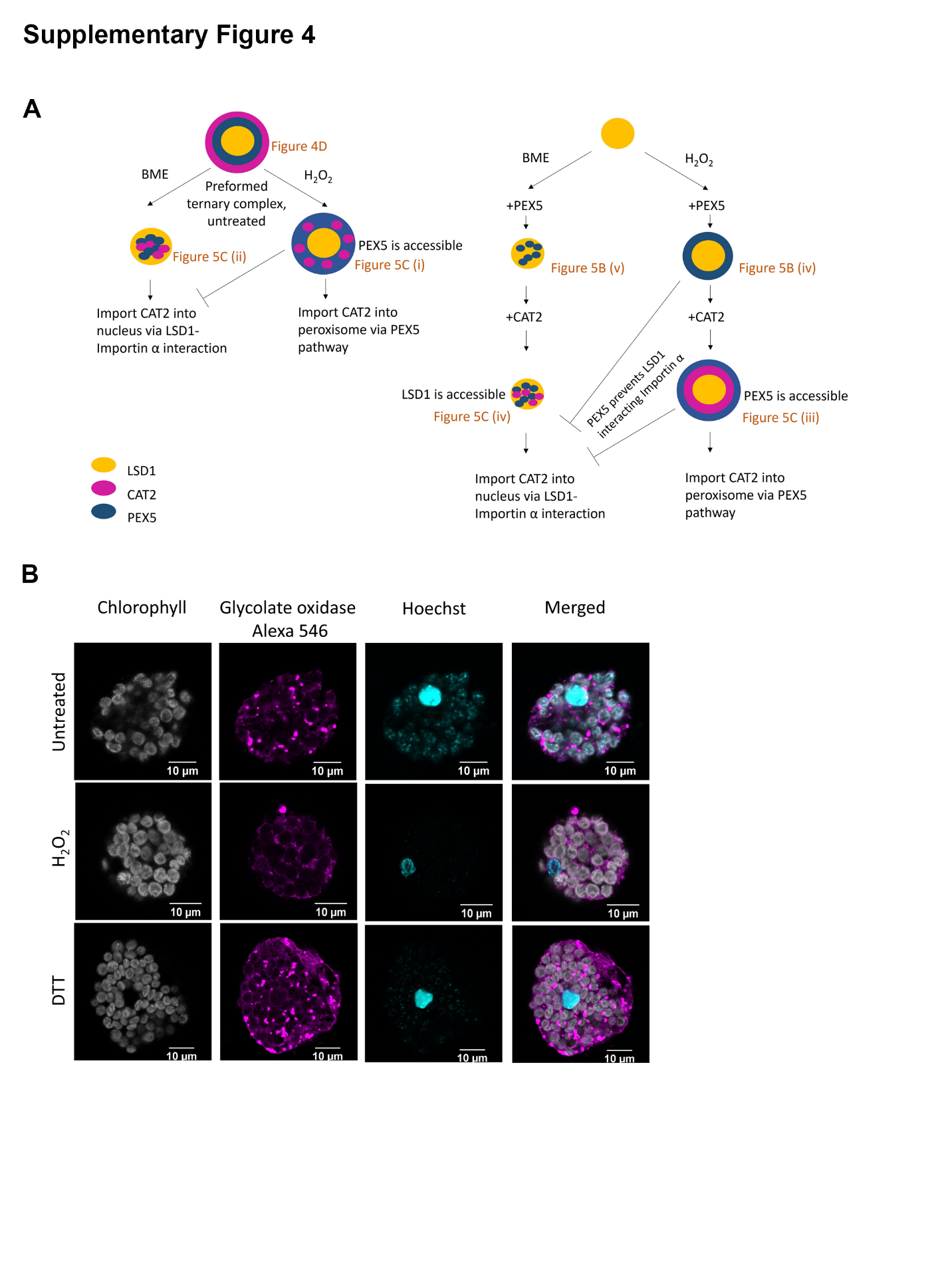

Supplement: Supplementary file 1 — Fig. S1 Production of recombinant Arabidopsis thaliana proteins used in this study. Fig. S2 Cellular expression and distribution of endogenous Arabidopsis thaliana LSD1. Fig. S3 Recombinant Arabidopsis thaliana LSD1 undergoes phase separation in vitro. Fig. S4 Effect of redox treatment on the localisation of Arabidopsis thaliana glycolate oxidase in protoplasts. Please note: Wiley is not responsible for the content or functionality of any Supporting Information supplied by the authors. Any queries (other than missing material) should be directed to the New Phytologist Central Office. [file NPH-247-2824-s001.docx]
